# The effects of exercise on circulating endocannabinoid levels—a protocol for a systematic review and meta-analysis

**DOI:** 10.1186/s13643-022-01980-x

**Published:** 2022-05-18

**Authors:** Viviane Bristot, Giorgio Poletto, Débora Maria Russiano Pereira, Melina Hauck, Ione Jayce Ceola Schneider, Aderbal S. Aguiar

**Affiliations:** 1 LABIOEX-Exercise Biology Lab, Department of Health Sciences, UFSC-Federal University of Santa Catarina, Araranguá, SC Brazil; 2 Sectorial Library of Campus Araranguá, UFSC-Federal University of Santa Catarina, Araranguá, SC Brazil; 3 LABEPI-Epidemiology Lab, Health Science Department and Rehabilitation Post-graduate Program, UFSC-Federal University of Santa Catarina, Araranguá, Santa Catarina Brazil

**Keywords:** Exercise, Physical activity, Endocannabinoids, Glyceryl 2-arachidonate, 2-Arachidonoylglycerol, Anandamide, n-Arachidonoylethanolamine, Endocannabinoid system, Endocannabinoid blood level, Systematic review

## Abstract

**Background:**

Increased circulating endocannabinoids levels are typically associated with aerobic exercise. This phenomenon is associated with a “runner’s high,” a state of euphoria and well-being experienced after a long exercise. We will provide in this review a transparent and standardized methodology following the PRISMA-P and Cochrane Handbook for Systematic Reviews of Interventions for conducting a systematic review and meta-analysis for synthesizing the available evidence about the effects of physical activity on the circulating levels of AEA and 2-AG endocannabinoids in healthy subjects.

**Methods:**

A multi-disciplinary team with basic and clinical expertise in exercise science developed this protocol. PubMed, EMBASE, Web of Science, CINAHL, SPORTDiscus, and Scopus will be the databases. A health sciences librarian was consulted in the development of the research. Search strategies will combine MeSH terms and free text words, including “exercise,” “exercise, physical,” “exercise training,” “physical activity,” “endocannabinoids,” “2-arachidonoyl-glycerol,” “glyceryl 2-arachidonate,” “2-AG,” “anandamide,” “AEA,” “n-arachidonoylethanolamide,” “adult,” “young adult,” and “middle-aged.” We will select experimental or quasi-experimental studies published through December 2021. The selection of studies, data extraction, assessment of the risk of bias, and the quality of evidence will be carried out in a paired and independent manner, and the consistency will be assessed using the statistics of Cohen Kappa. Methodological quality will be assessed using the Revised Cochrane risk of bias tool for randomized trials (RoB 2) and the Risk Of Bias In Nonrandomized Studies of Interventions (ROBINS-I) risk tool. We will use the Grading of Recommendations Assessment, Development, and Evaluation to assess the quality of the evidence, *χ*^2^ and *I*^2^ tests for heterogeneity, funnel plots, and the Egger test for publication bias. A meta-analysis for each data comparison will be performed whenever possible to determine the effect of physical activity on endocannabinoids’ circulating levels.

**Discussion:**

This systematic review and meta-analysis will provide an overview of the evidence about physical activity over AEA and 2-AG endocannabinoids, including comparability of variables between studies, critical interpretation of results, and use of accurate statistical techniques. The endocannabinoid is molecules by which muscles communicate with other tissues and organs, mediating the beneficial effects of exercise on health and performance, including increased glucose uptake, improved insulin action, and mitochondrial biogenesis. They are essential to exercise. Thus, this study will review the acute effect of physical exercise on circulating levels of endocannabinoids in healthy individuals. The results of this study will potentially be transferred to doctors, health professionals, and legislators to guide their decision making, as well as will improve future research.

**Systematic review registration:**

PROSPERO CRD42020202886.

**Supplementary Information:**

The online version contains supplementary material available at 10.1186/s13643-022-01980-x.

## Background

Anandamide or n-arachidonoylethanolamide (AEA) and 2-arachidonylglycerol (2-AG) are endogenous cannabinoids for the G-protein-coupled cannabinoid CB1 (type 1) and CB2 (type 2) receptors [1]. The endocannabinoid system plays a crucial role in the maintenance of homeostasis in thermoregulation and motor control [2], energy metabolism [3], skin function (barrier formation, regeneration) [4], appetite and digestion [5], learning and memory [6], chronic pain [7], and inflammation and other immune system responses [8]. Endurance running and cycling increase the circulating AEA and 2-AG produced by contracting muscles [9–12]. During/after a long run, AEA and 2-AG induce a state of euphoria, known as the “runner’s high” [13], which is related to the central effects of circulating endocannabinoids such as hedonic signal in the brain’s reward system known to encourage exercise usual aerobic. Additionally, the increase in endocannabinoid levels during exercise can also be a key element in incrementing brain-derived neurotrophic factor (BDNF), mediating benefits in cognition, as in neurogenesis and plasticity synaptic and antidepressant effects [11, 13]. These central effects added to the peripheral effects such as increased glucose absorption, better insulin action, and mitochondrial biogenesis, making it clinically and scientifically important to understand the response of endocannabinoids to exercise, especially the investigation of the exercise intensity necessary to produce such effects [11, 13]. .Functionally, these responses can be used therapeutically, for example, to improve performance and exercise capacity. However, there are controversies regarding the required exercise intensity to produce this effect [12, 14, 15]. Moreover, these effects are different for upper limb exercises. Long durations of arm exercises reduce circulating AEA, and resistance arm exercises do not affect 2-AG [14, 15].

Literature reviews have focused on the effect of physical activity on the endocannabinoid system and its impact on the pathology of neurological and neurodegenerative diseases, such as depression, anxiety, multiple sclerosis, epilepsy, Parkinson’s, and Alzheimer’s disease, in animal and human models [9, 16, 17]. They also addressed the benefits of exercise-induced endocannabinoid changes on brain function (cognition, mood, appetite, reward system), the musculoskeletal and adipose tissue (glucose regulation, insulin sensitivity, lipogenesis), and stress [18–20]. Moreover, Hillard [11] compiled information on circulating endocannabinoid levels and metabolic regulation, sleep, inflammation, and exercise. These were reviews of adaptations of the endocannabinoid system to physical activity with implications for health and well-being. These studies constitute the author’s interpretation and critical analysis of investigations of the endocannabinoid system as stimuli and potential sources of AEA and 2-AG present in circulation supported by citations and discussions. Unlike a systematic review that uses a methodology to gather all relevant evidence based on predefined eligibility criteria to answer a specific research question [21], statistical techniques such as meta-analysis can summarize the results to provide more accurate estimates of individual studies’ effects [21]. However, there are no reviews on the acute effects of physical activity on circulating endocannabinoids, a transient effect with significant biological effects. In this context, this protocol implies prior documentation to anticipate possible problems and improve planning before the review. A methodical and analytical approach will be employed. The acute and training effects (adaptations) of physical exercise are the two main exercise physiology objectives.

The purpose of this systematic review protocol is to provide a clear text and standardized study following the Preferred Reporting Items for Systematic reviews and Meta-Analysis Protocols (PRISMA-P) and Cochrane Handbook for Systematic Reviews of Interventions for conducting a systematic review and meta-analysis [21, 22]. The study’s primary outcome will be to review the effects of acute physical activity on circulating levels of AEA and 2-AG endocannabinoids in healthy individuals. The other outcomes are to identify the effects of different types of exercise [aerobic (prolonged duration), muscle endurance (short duration)], modalities (walking, running, cycling, free weights), and session duration (minutes). Besides, this systematic review will synthesize the best available evidence about acute physical activity and AEA and 2-AG endocannabinoids in this population.

## Methods/design

This protocol follows the PRISMA-P [21] and the Cochrane Handbook for Systematic Reviews of Interventions (Version 6) [22]. The PRISMA-P checklist can be found in Additional file 1. We registered in the International Prospective Register of Systematic Reviews (PROSPERO) (registration number: CRD42020202886). Any changes to this protocol will be described in the final review.

### Eligibility criteria

The eligibility criteria are under the Population-Intervention-Comparison-Outcomes-Study type (PICOS):

#### Population

We will include studies with individuals without diseases (healthy humans), young adults (18–35 years), and middle-aged adults (36–55), of any gender or ethnicity, and physically active (trained) or not trained [23]. Interventional studies with diseases and/or risk factors’ individuals in the intervention group will have included only control group individuals because this group has the eligibility criteria to be combined for synthesis [24].

Studies carried out with factors that can interfere with the reliability of the results, such as the use of psychotropic substances by population in the last 7 days (for example, synthetic cannabis/cannabis, cocaine, methamphetamine, ecstasy, and others) and the absence of relevant guidelines before the day of the test and/or intervention about food and exercise will be excluded.

#### Intervention

Studies will be eligible with at least one intervention group (physical activity or exercise), including a single session, regardless of its type (aerobic, muscular endurance), modality (walking, running, cycling, free weights), duration (week, months), frequency (days/week), session duration (minutes), number of sessions, prescription methods, or intensity [lactate, percentages of peak heart rate (peak HR), reserve HR (Karvonen), ventilatory thresholds in the cardiopulmonary test, percentage of power (Watt) and maximum repetition, subjective feeling of effort (Borg), others] [25].

No restrictions will be imposed regarding supervision (in person or not), the location of the intervention (clinic/health center, hospital, university, other), the type of performance (individual, group), or the specialization of the professionals who provided the physical activity or exercise (physiotherapist, fitness instructor, exercise scientist, other). Interventions that feature combined resources (for example, lifestyle change strategies, such as health education or diet/nutritional supplementation) will be excluded.

#### Comparison

The comparator will be a control group with individuals who did not participate in any form of intervention and/or a comparator group with baseline and immediately after intervention or recovery period measuring or concurrent intervention. Studies with control conditions that are not reported or cannot be calculated will be excluded.

#### Outcomes

We will include studies that evaluated circulating levels of AEA and 2-AG endocannabinoids immediately after the end of intervention (up to 5 and/or 10 min) without restriction to primary or secondary outcomes. Studies with measurements of salivary endocannabinoids or other endogenous ligands will not be included.

Studies that provide data from the same sample in two or more full-text will not be duplicated. We will consider only the study with the most prominent sample or present the most detailed results concerning its eligibility. This criterion will be defined by the team when analyzing the data extraction form; for example, the most detailed study will be the one that presents more information regarding the characteristics of the population, methods, and results.

#### Study type

We will include experimental studies (randomized and/or controlled clinical trials with at least one intervention group with physical activity or exercise and control group or intervention group with concurrent intervention) and/or quasi-experimental (clinical studies without control and/or comparator group). Observational studies and systematic reviews will be excluded.

### Search strategy

These are the electronic databases of the search: US National Library of Medicine (PubMed), EMBASE, Web of Science, Cumulative Index to Nursing and Allied Health Literature (CINAHL), SPORTDiscus, and Scopus, without language limitations and published through March 2021. The searches will be conducted again before the final analysis. Searches for unpublished studies (gray literature) will be conducted using OPEN GRAY, Networked Digital Library of Theses and Dissertation, and ProQuest to identify further information. The reference lists of the included studies and relevant reviews will also be analyzed manually, and specialists in the subject will also be identified and consulted to identify potential additional studies not included in the initial searches.

The search strategy will include different combinations based on medical subject headings terms and free text words and Boolean operators to ensure maximum capture of articles, including “exercise,” “exercise, physical,” “exercise training,” “physical activity,” “endocannabinoids,” “2-arachidonoyl-glycerol,” “glyceryl 2-arachidonate,” “2-AG,” “anandamide,” “AEA,” “n-arachidonoylethanolamide,” “adult,” “young adult,” and “middle-aged.” Extensive and sensitive literature searches without language restriction and searches in gray literature are strategies to prevent publication bias.

The preliminary search strategies were developed and tested in a pilot, adapted to each database (Additional file 2). The search strategy description includes all planned modifications to the indexing terms and free text words that vary between databases. A documentary librarian (DMRP) wrote and implemented the research strategy and assisted in selecting and evaluating the studies mentioned below.

### Selection of studies

The identified studies will be imported into reference management software (EndNote Web), and duplicate records will be deleted. Titles and abstracts will be evaluated and labeled agreements/disagreements on a specific platform for authors of systematic reviews (Rayyan) [26]. This study selection process will be carried out independently with blinding by two reviewers (VB and GP) familiar with the topic of interest to identify eligible studies. There will be no blinding to authors, institutions, or journals of the reviewed articles during the studies’ selection. Abstracts that do not provide sufficient information on the eligibility criteria will be selected for a more detailed evaluation by reading the article’s full text. The reviewers will then examine the articles to be included in a paired, independent, and blinded way, in the original format in full text, and available in full in each database. At this stage, studies considered irrelevant (according to the eligibility criteria listed above) will be excluded, and we will record the corresponding reason in the article selection flowchart. A third researcher (ASAJr) who will decide will resolve any inconsistencies between the reviewers regarding the selection. The selection process of the eligible studies shown in the PRISMA Flowchart provides an idea about the search strategy’s scope and increases the review’s internal validity.

### Data extraction

A data extraction form prepared in Microsoft Excel was explicitly developed for this review (Table 1). The extracted data will be filled in by two reviewers independently with blinding, including details about the publication, study design, characteristics of the population/intervention, and the results.

**Table 1:** Characteristics of the studies included in the systematic review and meta-analysis

				Intervention characteristics		Results
Publication details	Study design	Population characteristics		Physical activity or exercise		Circulating endocannabinoid levels
Title, first author, country of research, year of publication, journal, published language	Experimental (randomized or controlled clinical trials) or quasi-experimental studies	Source, sample size, eligibility criteria, sex (%), age (years, mean and standard deviation (total sample and/or by the group)	Physical activity level (trained/untrained), health condition	The number of participants in each group and type of intervention (anaerobic, aerobic, interval)	Protocol (intensity [lactate, percentages of peak heart rate (peak HR), reserve HR (Karvonen), ventilatory thresholds in the cardiopulmonary test, percentage of power (Watt) and maximum repetition, subjective feeling of effort (Borg), others], session duration (minutes), session frequency, intervention duration (weeks))	Summary data for each group, the difference between groups and/or change between baseline and post-intervention (mean and standard deviation, *P*-value, interquartile range, standard error and/or interval confidence)

After reading the included articles, additional data will be extracted when considered essential for the interpretation and applicability of the results, for example, the methods used for analyzing the endocannabinoids. In cases of missing data, the authors of the studies will be contacted by e-mail. If no response is received, the study will be excluded. Studies that meet the inclusion criteria will have the data extracted, like baseline values, immediately after the intervention and/or recovery period from the exercise values and the mean values and postintervention standard deviation. If available, the values of P, interquartile range (IQR), standard error, and interval confidence will be extracted.

### Risk of bias assessment

Randomized clinical trials will be evaluated using the Revised Cochrane risk-of-bias tool for randomized trials (RoB2) [27]. This tool covers the following criteria: randomization process, deviations from the intended interventions, missing results data, measurement of the results, selection of the reported result, and general bias. Each criterion will be evaluated and scored as low risk, some concerns, or a high risk of bias.

The Risk Of Bias In Nonrandomized Studies of Interventions (ROBINS-I) tool will assess the risk of bias in nonrandomized and quasi-experimental studies [28] will be used. It can be classified as without information, low risk, moderate, severe, or critical. The domains comprise confusion, selection of study participants, classification of interventions, deviations from intended interventions, missing data, measurement of results, and selecting the reported result. The risk of bias assessment will be carried out by two assessors independently, and any differences will be resolved by discussion and consensus.

### Data synthesis and analysis

The included studies will be stratified by type of intervention, and the results of aerobic training versus resistance training will be stratified/analyzed separately. Outcome measures will also be stratified and analyzed separately, based on the study design. The level of agreement between the two authors at each stage of the review (selection of studies, data extraction, assessment of the risk of bias, and quality of evidence) will be assessed using Cohen Kappa statistics. The closer this statistic is to 1, the higher the agreement among reviewers. The included studies will be summarized in an Ad Hoc table for comparison and assistance in critical interpretation. Whenever possible, we will use the results from an intention-to-treat analysis.

After data extraction, quantitative analysis by meta-analysis of the data will be performed considering the exercise type such as aerobic exercise and resistance exercise. The meta-analysis will also consider the type of aerobic (walking, running, or cycling) and resistance exercise (free weight or equipment). Besides, we will consider the exercise intensity (light, moderate or heavy). The results will be presented in tables and/or graphs that describe data such as means and standard deviation (from data calculated), the effect size as a standardized mean difference with 95% CI and study weighting. The forest plots will be created using Review Manager software (RevMan 5.4) [29] to illustrate the individual and grouped effect sizes. The standardized mean difference will be calculated as the difference in means between the values (baseline/postintervention and/or intervention/control) divided by the pooled standard deviation of the measurements. For this study, the effect size will be categorized as follows: small (ranging from 0 to 0.2), moderate (ranging from 0.2 to 0.5), and large (ranging from 0.5 to 0.8). The studies’ weighting effect will be calculated using the inverse variance method (individual effect sizes multiplied by the inverse of their standard squared error) to reflect individual studies’ contribution to the total effect estimate.

The heterogeneity assessment will include the *χ*^2^ test to verify heterogeneity, with a significance level of *p* < 0.10. The magnitude of the heterogeneity of each meta-analysis will be quantified by the *I*^*2*^ statistic, interpreted as follows: might not be essential (0– 40%), moderate (30– 60%), substantial (50– 90%), and considerable (75– 100%). If *I*^*2*^ is ≤50%, the fixed-effects model (Mantel-Haenszel method) will be used for the meta-analysis. Although an *I*^*2*^ > 50% represents heterogeneity, the random-effects model (DerSimonian and Laird method) will collect the results. Considering the possibility of high heterogeneity, we will analyze its possible sources to obtain an objective conclusion.

The analysis of the source of heterogeneity (sensitivity, subgroup, and/or meta-regression analysis) will be defined only during the review process, where the peculiarities of the included studies will be identified, such as sample size, sex, age, overload principle (intensity versus volume), adaptation to exercise, randomization, missing data, and other relevant situations.

The funnel plot will be used to assess publication bias, and the Egger test will check the asymmetry of plotting the funnel. Asymmetric funnel plots indicate publication bias, which is a reporting bias, but it also implies that there may be other causes, such as differences in methodological quality or heterogeneous effects of the intervention. We will review the possible reasons and explain any asymmetric funnel plot.

### Assessment of the quality of the evidence

The quality of the evidence will be analyzed in a paired and independent way using the Grading of Recommendations Assessment, Development, and Evaluation (GRADE) [30, 31]. The GRADE tool consists of the following criteria: methodological limitations (risk of bias), inconsistent results, indirect evidence, imprecision, and publication bias. For each analyzed result, the evidence’s quality will be classified as very low, low, moderate, or high using the GRADE profiler (GRADEpro) Guideline Development Tool [32]. Disagreements will be resolved by discussion and consensus. The results will be presented in a “Summary of Findings” table.

## Discussion

This review will be the first to systematically identify and analyze evidence on the acute effects of physical activity on circulating levels of endocannabinoids like AEA and 2-AG. Levels of AEA and 2-AG are considered a physiological response or acute effect of physical activity or exercise. The World Health Organization [23] defines physical activity as “anybody movement produced by skeletal muscles that require energy expenditure.” Exercise is defined as planned, structured, regular physical activity aiming to improve or maintain physical fitness.

The assessment of the risk of bias, the quality of evidence, and heterogeneity, with particular reference to the sample’s characteristics, is a point highlighted in this review. This protocol presents an explicit and replicable methodology through the search strategy, study selection, and data extraction, in addition to synthesizing the data using meta-analyses. This study will provide an overview of the evidence available to avoid duplication of research. It will also identify possible knowledge gaps and inform experimental protocols in physical activity and endocannabinoid research.

The results of this study will be helpful to guide the design of future clinical and scientific interventions aimed at the effectiveness of physical exercise on the endocannabinoid system, especially for recommending the type of exercise and intensity that increase the response endocannabinoids to mediate beneficial effects on health and performance. Above all, this study’s results will provide the highest level of evidence that can potentially be transferred to clinicians, healthcare professionals, and policymakers to guide their decision-making.

## Supplementary Information

**Additional file 1.**

**Additional file 2.**

## Data Availability

The datasets used and/or analyzed during the current study are available from the corresponding author on reasonable request.

## Abbreviations

AEA: n-Arachidonylethanolamide
2-AG: 2-Arachidonylglycerol
CB1: Cannabinoid receptors type 1
CB2: Cannabinoid receptors type 2
PRISMA-P: Preferred Reporting Items for Systematic reviews and Meta-Analysis Protocols
PROSPERO: International Prospective Register of Systematic Reviews
PICOS: Population-Intervention-Comparison-Outcomes-Study type
HR: Heart rate
PubMed: US National Library of Medicine
CINAHL: Cumulative Index to Nursing and Allied Health Literature
IQR: Interquartile range
RoB2: Revised Cochrane risk-of-bias tool for randomized trials
ROBINS-1: Risk Of Bias In Nonrandomized Studies of Interventions
RevMan: Review Manager
GRADE: Grading of Recommendations Assessment, Development, and Evaluation
GRADEpro: Grading of Recommendations Assessment, Development, and Evaluation profiler
