# Supplementary material for: The effects of exercise on circulating endocannabinoid levels—a protocol for a systematic review and meta-analysis
Source: Syst Rev. 2022 May 18;11:98. doi: 10.1186/s13643-022-01980-x (PMC9115961; doi:10.1186/s13643-022-01980-x)
Supplement: Supplementary file 2 — Additional file 2. [file 13643_2022_1980_MOESM2_ESM.docx]

**ADDITIONAL FILE 2**

The search strategies and pilots tested in each database are listed below:

**Database 1: PubMed**

#1

"Exercise"[MeSH Terms] OR "Exercise"[Title/Abstract] OR "Exercises"[Title/Abstract] OR "Physical Activity"[Title/Abstract] OR "activities physical"[Title/Abstract] OR "activity physical"[Title/Abstract] OR "Physical Activities"[Title/Abstract] OR "exercise physical"[Title/Abstract] OR "exercises physical"[Title/Abstract] OR "Physical Exercise"[Title/Abstract] OR "Physical Exercises"[Title/Abstract] OR "Acute Exercise"[Title/Abstract] OR "Acute Exercises"[Title/Abstract] OR "exercise acute"[Title/Abstract] OR "exercises acute"[Title/Abstract] OR "exercise isometric"[Title/Abstract] OR "exercises isometric"[Title/Abstract] OR "Isometric Exercises"[Title/Abstract] OR "Isometric Exercise"[Title/Abstract] OR "exercise aerobic"[Title/Abstract] OR "Aerobic Exercise"[Title/Abstract] OR "Aerobic Exercises"[Title/Abstract] OR "exercises aerobic"[Title/Abstract] OR "Exercise Training"[Title/Abstract] OR "Exercise Trainings"[Title/Abstract] OR "training exercise"[Title/Abstract]

#2

"Endocannabinoids"[MeSH Terms] OR "Endocannabinoids"[Title/Abstract] OR "Endocannabinoid"[Title/Abstract] OR "2-arachidonoyl-glycerol"[Title/Abstract] OR "2-arachidonyl-glycerol"[Title/Abstract] OR "2-arachidonylglycerol"[Title/Abstract] OR "2-arachidonoylglycerol"[Title/Abstract] OR "2-AG"[Title/Abstract] OR "anandamide"[Title/Abstract] OR "n 2 hydroxyethyl arachidonamide"[Title/Abstract] OR "arachidonoyl ethanolamide"[Title/Abstract] OR "arachidonoylethanolamide"[Title/Abstract] OR "arachidonylethanolamide"[Title/Abstract] OR "5 8 11 14 eicosatetraenamide n 2 hydroxyethyl"[Title/Abstract] OR "anandamide 20 4 n 6"[Title/Abstract] OR "n-arachidonoylethanolamide"[Title/Abstract] OR "AEA"[Title/Abstract]

#3

"Adult"[MeSH Terms] OR "Adult"[Title/Abstract] OR "Adults"[Title/Abstract] OR "Young Adult"[MeSH Terms] OR "Young Adult"[Title/Abstract] OR "adult young"[Title/Abstract] OR "adults young"[Title/Abstract] OR "Young Adults"[Title/Abstract] OR "Middle Aged"[MeSH Terms] OR "Middle Aged"[Title/Abstract] OR "Middle Age"[Title/Abstract]

**Database 2: EMBASE**

#1

'exercise'/exp OR exercise:ab,ti OR 'aerobic exercise'/exp OR 'aerobic exercise':ab,ti OR 'exercise training':ab,ti OR 'exercise, aerobic':ab,ti OR 'exercise, isometric':ab,ti OR 'physical exercise':ab,ti OR 'isometric exercise'/exp OR 'isometric exercise':ab,ti OR 'physical activity'/exp OR 'physical activity':ab,ti OR 'activity, physical':ab,ti OR 'anaerobic exercise'/exp OR 'anaerobic exercise':ab,ti OR 'endurance training'/exp OR 'endurance training':ab,ti OR 'isokinetic exercise'/exp OR 'isokinetic exercise':ab,ti OR 'isotonic exercise'/exp OR 'isotonic exercise':ab,ti OR 'resistance training'/exp OR 'resistance training':ab,ti

#2

'endocannabinoid'/exp OR endocannabinoid:ab,ti OR endocannabinoids:ab,ti OR 'endogenous cannabinoid':ab,ti OR 'endocannabinoid system'/exp OR 'endocannabinoid system':ab,ti OR 'endocannabinoid 2 arachidonoylglycerol'/exp OR 'endocannabinoid 2 arachidonoylglycerol':ab,ti OR 'endocannabinoid blood level'/exp OR 'endocannabinoid blood level':ab,ti OR '2 arachidonoylglycerol'/exp OR '2 arachidonoylglycerol':ab,ti OR '2-arachidonylglycerol':ab,ti OR 'glyceryl 2 arachidonate'/exp OR 'glyceryl 2 arachidonate':ab,ti OR '2 arachidonoyl glycerol'/exp OR '2 arachidonoyl glycerol':ab,ti OR 'anandamide'/exp OR anandamide:ab,ti OR arachidonoylethanolamide:ab,ti OR arachidonylethanolamide:ab,ti OR (n:ab,ti AND '2 hydroxyethyl':ab,ti AND arachidonamide:ab,ti) OR 'n arachidonoylethanolamine':ab,ti

#3

'young adult'/exp OR 'young adult':ab,ti OR 'adult'/exp OR adult:ab,ti OR 'middle ages'/exp OR 'middle ages':ab,ti

**Database 3: Web of Science - Main Collection (Clarivate Analytics).**

#1

TS=("Exercise" OR "Exercises" OR "Physical Activity" OR "Activities, Physical" OR "Activity, Physical" OR "Physical Activities" OR "Exercise, Physical" OR "Exercises, Physical" OR "Physical Exercise" OR "Physical Exercises" OR "Acute Exercise" OR "Acute Exercises" OR "Exercise, Acute" OR "Exercises, Acute" OR "Exercise, Isometric" OR "Exercises, Isometric" OR "Isometric Exercises" OR "Isometric Exercise" OR "Exercise, Aerobic" OR "Aerobic Exercise" OR "Aerobic Exercises" OR "Exercises, Aerobic" OR "Exercise Training" OR "Exercise Trainings" OR "Training, Exercise" OR "Trainings, Exercise")

#2

TS=("Endocannabinoids" OR "Endocannabinoid" OR "glyceryl 2-arachidonate" OR "2-arachidonoyl-glycerol" OR "2-monoarachidonoylglycerol" OR "2-arachidonyl-glycerol" OR "2-arachidonylglycerol" OR "5,8,11,14-eicosatetraenoic acid, 2-hydroxy-1-(hydroxymethyl)ethyl ester, (all-Z)-" OR "2-arachidonoylglycerol" OR "2-AG" OR "anandamide" OR "5,8,11,14-eicosatetraenoylethanolamide" OR "N-(2-hydroxyethyl)arachidonamide" OR "N-arachidonoyl-2-hydroxyethylamide" OR "arachidonoyl ethanolamide" OR "arachidonoylethanolamide" OR "arachidonylethanolamide" OR "5,8,11,14-eicosatetraenamide, N-(2-hydroxyethyl)-" OR "anandamide (20.4,n-6)" OR "n-arachidonoylethanolamide" OR "AEA")

#3

TS=("Adult" OR "Adults" OR "Young Adult" OR "Adult, Young" OR "Adults, Young" OR "Young Adults" OR "Middle Aged" OR "Middle Age")

**Database 4: CINAHL with Full Text (EBSCO).**

1#

MH "Exercise" OR MH "Resistance Training" OR MH "Aerobic Exercises" OR TX "Aerobic Exercise" OR TX "Physical Exercise" OR TX "Exercise training" OR MH "Isometric Exercises" OR TX "Exercise, Isometric" OR TX "Exercises, Isometric" OR TX "Physical Exercise" OR MH "Physical Activity" OR TX "Physical Activities"

#2

TX "Endocannabinoid" OR TX "Endocannabinoids" OR TX "Endogenous cannabinoid OR TX "Endocannabinoid system" OR TX "Endocannabinoid 2 arachidonoylglycerol" OR TX "Endocannabinoid blood level" OR TX "Glyceryl 2-arachidonate" OR TX "2-arachidonoyl-glycerol" OR TX "2-AG" OR TX "Anandamide" OR TX "AEA" OR TX "n-arachidonoylethanolamide"

#3

MH "Adult" OR TX "Adults" OR MH "Young Adult" OR TX "Adult, Young" OR TX "Adults, Young" OR TX "Young Adults" OR MH "Middle Aged" OR TX "Middle Age"

**Database 5: SPORTDiscus with Full Text (EBSCO).**

S1

TX "Exercise" OR TX "Resistance training" OR TX "Aerobic exercises" OR TX "Physical exercise" OR TX "Anaerobic exercises" OR TX "Isometric exercise" OR TX "Isotonic exercise" OR TX "Isokinetic exercise" OR TX "Physical activity" OR TX "Strength training" OR TX "Physical Activity"

S2

TX "endocannabinoid" OR TX "endocannabinoids" OR TX "endogenous cannabinoid" OR TX "endocannabinoid system" OR TX "endocannabinoid 2 arachidonoylglycerol" OR TX "endocannabinoid blood level" OR TX "glyceryl 2-arachidonate" OR TX "2-arachidonoyl-glycerol" OR TX "2-AG" OR TX "Anandamide" OR TX "AEA" OR TX "n-arachidonoylethanolamide"

S3

TX "Adult" OR TX "Adults" OR TX "Young Adult" OR TX "Adult, Young" OR TX "Adults, Young" OR TX "Young Adults" OR TX "Middle Aged" OR TX "Middle Age"

**Database 6: Scopus.**

#1

TITLE-ABS-KEY ("Exercise" OR "Exercises" OR "Physical Activity" OR "Activities, Physical" OR "Activity, Physical" OR "Physical Activities" OR "Exercise, Physical" OR "Exercises, Physical" OR "Physical Exercise" OR "Physical Exercises" OR "Acute Exercise") OR TITLE-ABS-KEY ( "Acute Exercises" OR "Exercise, Acute" OR "Exercises, Acute" OR "Exercise, Isometric" OR"Exercises, Isometric" OR "Isometric Exercises" OR "Isometric Exercise" OR "Exercise, Aerobic" OR "Aerobic Exercise" OR "Aerobic Exercises" OR "Exercises, Aerobic" ) OR TITLE-ABS-KEY ( "Exercise Training" OR "Exercise Trainings" OR "Training, Exercise" OR "Trainings, Exercise")

#2

TITLE-ABS-KEY ("Endocannabinoids" OR "Endocannabinoid" OR "glyceryl 2-arachidonate" OR "2-arachidonoyl-glycerol" OR "2-monoarachidonoylglycerol" OR "2-arachidonyl-glycerol" OR "2-arachidonylglycerol") OR TITLE-ABS-KEY ("5,8,11,14-eicosatetraenoic acid, 2-hydroxy-1-(hydroxymethyl)ethyl ester, (all-Z)-" OR "2-arachidonoylglycerol" OR "2-AG" OR "anandamide" OR "5,8,11,14-eicosatetraenoylethanolamide" OR "N-(2-hydroxyethyl)arachidonamide" ) OR TITLE-ABS-KEY ("N-arachidonoyl-2-hydroxyethylamide" OR "arachidonoyl ethanolamide" OR "arachidonoylethanolamide" OR "arachidonylethanolamide" OR "5,8,11,14-eicosatetraenamide, N-(2-hydroxyethyl)-" OR "anandamide (20.4,n-6)" OR "n-arachidonoylethanolamide" OR "AEA")

#3

TITLE-ABS-KEY ("Adult" OR "Adults" OR "Young Adult" OR "Adult, Young" OR "Adults, Young" OR "Young Adults" OR "Middle Aged" OR "Middle Age")
